# A Forum for a Highly Important and Ever-Expanding Field of Study

**DOI:** 10.1155/PPAR/2006/61385

**Published:** 2006-06-19

**Authors:** Mostafa Badr

**Affiliations:** Division of Pharmacology, School of Pharmacy, University of Missouri-Kansas City, 2411 Holmes Street, Kansas City, MO 64108, USA

*Peroxisome Proliferator-Activated Receptors* (PPARs) were discovered by Isseman and Green in 1990 [1]. Since this time PPARs have been demonstrated to play a major role in a diverse group of processes and pathological conditions associated with aging [2, 3], inflammation [4, 5], immunity [6], obesity [7], diabetes [8], cancer [9], and fertility [10, 11].

In the short period since their discovery, interest in the functioning of the PPAR receptors has greatly intensified, and studies of the biology of the PPARs have become abundant. Accordingly, the number of publications in this field has undergone exponential growth, climbing from less than 100 published articles in 1995 to more than 1600 articles in 2005; continued growth is to be expected. Of further importance, the PPARs have entered directly into the clinical arena. Drugs which activate PPARs are currently used clinically to treat diabetes, and others are also undergoing clinical trials as anti-inflammatory agents.

*PPAR Research* is designed to be a much needed, open access forum dedicated to the publication of original, high-quality, peer-reviewed articles on the advances in basic research, preclinical studies, and clinical trials involving PPARs, and their obligatory heterodimer partners, retinoid-X receptors. This Journal will provide a unique venue to bring together a diverse scope of studies and will offer to both scientists and clinicians working in this field of study the opportunity to learn of the latest discoveries in areas other than their own.

*PPAR Research* also intends to illustrate the magnitude and significance of this discipline by bringing into focus the diversity of areas of study involved in PPAR research. This will surely promote interest in as well as enhanced understanding of this critical field of research by the scientific community at large.
